# Multidimensional poverty and the co-occurrence of undernutrition and intestinal parasitic infections in Ecuadorian infants: a geospatial analysis

**DOI:** 10.3389/fpubh.2025.1668303

**Published:** 2025-11-19

**Authors:** Pamela Vinueza-Veloz, Andrés Fernando Vinueza-Veloz, Estephany Tapia-Veloz, Gabriela Tapia-Veloz, Tannia Valeria Carpio-Arias, Maria Fernanda Vinueza-Veloz

**Affiliations:** 1 Grupo de Investigación en Ciencias Veterinarias, Escuela Superior Politécnica de Chimborazo, Riobamba, Ecuador; 2 Research Group on Human Food and Nutrition (GIANH), Escuela Superior Politécnica de Chimborazo, Riobamba, Ecuador; 3 Department of Pharmacy, School of Health Sciences, Universidad Cardenal Herrera-CEU, CEU Universities, Valencia, Spain; 4 Area of Parasitology, Department of Pharmacy and Pharmaceutical Technology and Parasitology, University of Valencia, Valencia, Spain; 5 Department of Community Medicine and Global Health, Institute of Health and Society, University of Oslo, Oslo, Norway

**Keywords:** poverty, undernutrition, intestinal parasitic infection, spatial analysis, Ecuador

## Abstract

**Objective:**

This study investigated the relationship between multidimensional poverty and the co-occurrence of undernutrition and intestinal parasitic infections among children under five in Ecuador.

**Materials and methods:**

Using the 2021 national outpatient Ecuadorian registry, we assessed the co-occurrence of undernutrition and intestinal parasitic infection incidences at the municipal-level through spatial analysis. We applied spatial autocorrelation to identify geographic co-occurrence clusters of high-high and low-low incidence rates. Subsequently, we compared the proportions of multidimensional poverty between the high-high and low-low co-occurrence clusters.

**Results:**

A spatial correlation was observed between the incidence of undernutrition (14.57 per 1,000 child-years) and intestinal parasitic infections (34.07 per 1,000 child-years) (Bivariate Moran’s Index = 0.19, *p* < 0.001). High-high incidence clusters for both conditions were concentrated in the Amazon region. The median multidimensional poverty in these high-high incidence clusters was 72.62% (IQR: 62.08–82.44%), which was nearly twice as high as in low-low incidence clusters (38.15%; IQR: 38.15–38.93%).

**Conclusion:**

Our findings suggest that undernutrition and intestinal parasitic infections in children under 5 years of age tend to co-cluster, and their joint occurrence serves as an indicator of social inequity. High-high incidence clusters were concentrated in the nation’s poorest regions, particularly the Amazon. To overcome this double burden, public-health measures must go beyond biomedical interventions and address its underlying social and structural determinants.

## 1 Introduction

Child undernutrition remains a major global public health concern, particularly in low- and middle-income countries (1). It is associated with increased childhood mortality and morbidity, largely due to heightened susceptibility to infections (2). Beyond its immediate health effects, undernutrition has long-term consequences, including an elevated risk of non-communicable diseases in adulthood (3) Worldwide, an estimated 23% of children under five experience linear growth failure (stunting) and approximately 7% suffer from wasting, a severe form of acute undernutrition (1) The burden is especially pronounced in Ecuador, where 23% of children under five are chronically undernourished—the second-highest prevalence in Latin America, surpassed only by Guatemala (39%) (1, 4).

Intestinal parasitic infections are particularly prevalent among undernourished children and play a critical role in exacerbating nutritional deficits, thereby perpetuating a self-reinforcing cycle of undernutrition and infection (5). The intestinal parasites most frequently reported in undernourished children include *Ascaris lumbricoides* (roundworm), hookworms (*Ancylostoma duodenale, Necator americanus*), *Trichuris trichiura* (whipworm), and *Giardia duodenalis* (5, 6). In children under five, infection with *A. lumbricoides* is associated with a higher risk of stunting (OR: 2.17; 95% CI: 1.14–4.13), while infection with *G. duodenalis* with increased odds of underweight (OR: 1.53; 95% CI: 1.02–2.29) and wasting (OR: 2.90; 95% CI: 1.12–7.49) (5).

In Ecuador, a recent study that used molecular diagnostic techniques confirmed a substantial parasitic burden among children (7). The authors found *G. duodenalis* to be the most prevalent intestinal parasite, followed by helminths (*T. trichiura, A. lumbricoides, Strongyloides stercoralis*). Notably, the combined prevalence of protozoa and helminths exceeded 50% in several regions, underscoring the magnitude of the problem (7). Consistent with global evidence, Ecuadorian investigations have also shown that children infected with intestinal parasites are more likely to experience stunting and underweight compared to their non-infected peers (8, 9).

Childhood undernutrition and intestinal parasitic infections disproportionately affect populations in low-resource settings, where poverty perpetuates their co-occurrence (10, 11). This complex interplay can be conceptualized as a *syndemic*— a synergistic clustering of multiple health conditions in a population, intensified by social and structural determinants (12). The syndemic framework posits that such conditions cluster and interact, worsening the overall health burden beyond the sum of their individual effects. The high prevalence and known biological synergy between these conditions in Ecuador suggest that such a syndemic process may be occurring, disproportionately impacting marginalized populations.

While direct evidence for the existence of a syndemic process is complex and limited, a critical first step is to identify whether the necessary population-level preconditions exist: namely, the geographic co-clustering of these conditions with areas of high multidimensional poverty (13). Although earlier Ecuadorian studies have examined undernutrition and intestinal parasitic infections separately, few have investigated their co-occurrence or shared determinants (14, 15). Therefore, the present study aims to investigate the relationship between multidimensional poverty and the co-occurrence of child undernutrition and intestinal parasitic infections in Ecuador, using an ecological and spatial analysis approach.

## 2 Materials and methods

### 2.1 Study design and geographic context

This study applied an ecological and cross-sectional design. It was conducted in Ecuador, a country located in northwestern South America, bordered by Colombia and Peru. Geographically, Ecuador is divided into four regions: the Coast (Pacific littoral), Highlands (Andean mountains), Amazon (tropical rainforest), and the Insular Region (Galápagos Islands) (Supplementary material 1). As of 2021, Ecuador had an estimated under-five population of 1.6 million children. Administratively, the country is divided into 221 municipalities, which were the units of analysis (16).

### 2.2 Data sources and variables

#### 2.2.1 Data sources

Data on cases of undernutrition and intestinal parasitic infections among children under five years of age in 2021 were retrieved from the national outpatient consultation registry *Consultas Ambulatorias-2021*. The database is compiled and published by the *Ministerio de Salud Pública del Ecuador* and is publicly accessible via https://datosabiertos.gob.ec/dataset/https-almacenamiento-msp-gob-ec-index-php-s-n5kn91dvcgpskpd. For each medical consultation, the database contains demographic information and diagnoses obtained during care, which are coded in the International Classification of Diseases, 10^th^ Revision (ICD-10) format from public clinics within Ecuador’s national health system.

Population denominator data for children under 5 years of age (2021) are derived from population projections based on the 2022 census conducted by *Instituto Nacional de Estadísticas y Censos* (INEC). Municipal-level proportions of multidimensional poverty for 2021 (PMP), estimated by the INEC were sourced from the annual *Encuesta Nacional de Empleo, Desempleo y Subempleo* repository, available at https://www.ecuadorencifras.gob.ec/enemdu-anual-2021/. Municipal polygons were obtained from INEC geospatial repository, accessible at https://www.ecuadorencifras.gob.ec/documentos/web-inec/Geografia_Estadistica/Micrositio_geoportal/index.html.

#### 2.2.2 Case definition

Cases of undernutrition were identified through specific ICD-10 codes related to protein-energy deficiency, namely E40 (Kwashiorkor), E41 (Nutritional marasmus), E42 (Marasmic kwashiorkor), E43 (Unspecified severe protein-calorie deficiency), E44.0 (Moderate protein-calorie deficiency), E44.1 (Mild protein-calorie deficiency), and E46 (Unspecified protein-calorie deficiency). For the purpose of descriptive analyses, undernutrition cases were classified in four groups: Mild (E44.1), moderate (E44.0), severe (E40, E41, E42, E43) and unspecified (E46). Intestinal parasitic infections were identified using ICD-10 codes for gastrointestinal parasites (Supplementary material 2). The vast majority of cases (94%) were recorded under the code B83.9 (unspecified helminthiasis). For cases with a specified pathogen in the diagnosis (<5% of records), we classified the parasite at the family level using a standardized taxonomic hierarchy (e.g., Metazoa, Nematoda, Rhabditida, Ascarididae, *A. lumbricoides*).

#### 2.2.3 Incidence rates of undernutrition and intestinal parasitic infections

Municipal-level cases of undernutrition and intestinal parasitic infections were aggregated by the children’s usual place of residence. The number of cases was divided by the corresponding municipality’s population of children under 5 years of age to calculate crude incidence rates, expressed as cases per 1,000 child-years. Poisson regression was used to estimate 95% confidence intervals. To address instability in rate estimation caused by small population denominators, empirical Bayesian smoothing was applied, adjusting observed incidence rates toward a global prior mean to reduce variance. Three municipalities (El Piedrero, Las Golondrinas, and Manga del Cura) were excluded from the analysis due to territorial jurisdictional disputes and absence of registered cases, resulting in a final analytical sample of 218 municipalities. Cases from the Insular Region (representing 0.1% of the national under-five population) were combined with the Coast Region to compute regional incidence rates.

#### 2.2.4 Multidimensional poverty

This variable expresses the percentage of households within a province that are classified as multidimensionally poor, meaning they experience simultaneous deprivations in at least 33.3% of the indicators across four dimensions of well-being. These dimensions include: (i) Education (school non-attendance, financial barriers to university, incomplete basic education); (ii) employment and social security (child labor, unemployment or underemployment, lack of pension contributions); (iii) health, water, and nutrition (extreme income poverty, absence of sewage systems); and (iv) habitat (overcrowding, substandard housing, lack of sanitation or waste collection) (17, 18).

### 2.3 Statistical analysis

#### 2.3.1 Descriptive analysis

Records lacking residential data were excluded to ensure geographic consistency. Analyses were stratified by sex (female/male; assigned at birth). Age distributions between groups were compared using the Wilcoxon test due to non-normal distributions (Kolmogorov–Smirnov: undernutrition cases, *D* = 0.20, *p* < 0.010; parasitic infections, *D* = 0.50, *p* < 0.01). Associations between undernutrition severity and parasitic infection taxonomies were assessed via Pearson’s chi-square test. The spatial distribution of diseases was described using crude incidence rates, visualized through thematic maps that employed municipal polygons as the analytical units.

#### 2.3.2 Spatial data analysis

Galápagos was excluded from all spatial analyses due to its pronounced geographical isolation (>1,000 km from mainland Ecuador), which violates the principle of spatial contiguity required for a queen-contiguity-based spatial weights matrix. The analysis therefore proceeded using empirical Bayesian-smoothed incidence rates from the 218 continental municipalities in two sequential stages. First, we assessed global spatial autocorrelation via the Global Moran’s Index, which revealed autocorrelation (Index = 0.460, *p <* 0.001) suggesting clustering. Local spatial clusters were identified using Local Indicators of Spatial Association (LISA) following Anselin’s methodology (19). Hotspots denote geographically concentrated areas of high incidence rates. These were operationally defined as municipalities meeting three criteria: (i) statistically significant positive local Moran’s Index values; (ii) incidence rates exceeding the national mean; and (iii) spatial adjacency to municipalities exhibiting similarly elevated rates. In contrast, coldspots denote geographically concentrated areas of low incidence rates. Coldspots were defined as municipalities with (i) statistically significant positive local Moran’s Index values; (ii) incidence rates below the national mean; and (iii) spatial adjacency to municipalities exhibiting similarly low rates (19). Second, we employed the bivariate Moran’s Index to identify municipalities with spatial similarity in the incidence rates of undernutrition and intestinal parasitic infections. This analysis classified areas as high-high clusters (co-occurring hot spots) or low-low clusters (co-occurring cold spots). Statistical significance for these bivariate associations was evaluated using Monte Carlo randomization with 999 permutations and a fixed random seed (GeoDa seed = 1,234).

#### 2.3.3 Comparison between clusters

After confirming a non-normal distribution of multidimensional poverty values via the Shapiro–Wilk test (W = 0.80, *p <* 0.001), we used the Wilcoxon rank-sum test to compare multidimensional poverty values between the identified high-high and low-low clusters.

### 2.4 Ethical considerations and bias control

This study analyzed publicly available, anonymized datasets from INEC. Ethical approval was not required, as the research used aggregated, de-identified data under open-access policies. The project followed institutional ethical guidelines for secondary data analysis and was conducted under *Escuela Superior Politécnica de Chimborazo’s GUAGUA* research initiative (Approval Code: IDIPI-257).

## 3 Results

In 2021, Ecuador recorded 17,493,594 outpatient consultations, including 24,490 undernutrition and 57,554 intestinal parasitic infections registries. After excluding 38 undernutrition and 330 intestinal parasitic infection cases with missing residence data, the analysis included 57,224 and 24,452 cases, respectively. In 2021 the under-five population was estimated at 1,678,800.

### 3.1 Undernutrition

The median age of undernutrition cases was 1 year (interquartile range, IQR: 0–4). Male children accounted for 55.40% of cases, while female children represented 44.60%. There was a significant difference between age of diagnosis among male and female children, but this was small and likely not clinically meaningful (Table 1). Mild undernutrition cases predominated (31.00%), followed by unspecified (30.42%) and moderate (28.13%). Severe cases represented 10.45% of reported undernutrition cases. Male children showed slightly higher percentages of unspecified, moderate and severe cases (Table 1).

**Table 1:** Characterization of undernutrition cases in Ecuadorian children under five years, 2021.

	Overall	Female	Male	Statistical test
	24,452 (100)	10,927 (44.60)	13,525 (55.40)	
Age, median (IQR)	1 (0–2)	1 (0–2)	1 (0–2)	Wilcoxon, *p* < 0.001
Severity				
Mild, *n* (%)	7,580 (31.00)	3,492 (31.96)	4,088 (30.23)	Chisq. (3 df) = 10.51, *p <* 0.001
Moderate, *n* (%)	6,878 (28.13)	3,050 (27.91)	3,828 (28.30)	
Unspecified, *n* (%)	7,439 (30.42)	3,292 (30.13)	4,147 (30.66)	
Severe, *n* (%)	2,555 (10.45)	1,093 (10.00)	1,462 (10.81)	

Case data were obtained from the 2021 national outpatient consultation registry of the Ministerio de Salud Pública del Ecuador (see Methods). Cases were then classified into four groups according to their ICD‑10 codes, as described in the Methods. IQR, interquartile range; n, number; %, percentage; df, degrees of freedom.

**Figure 1:**
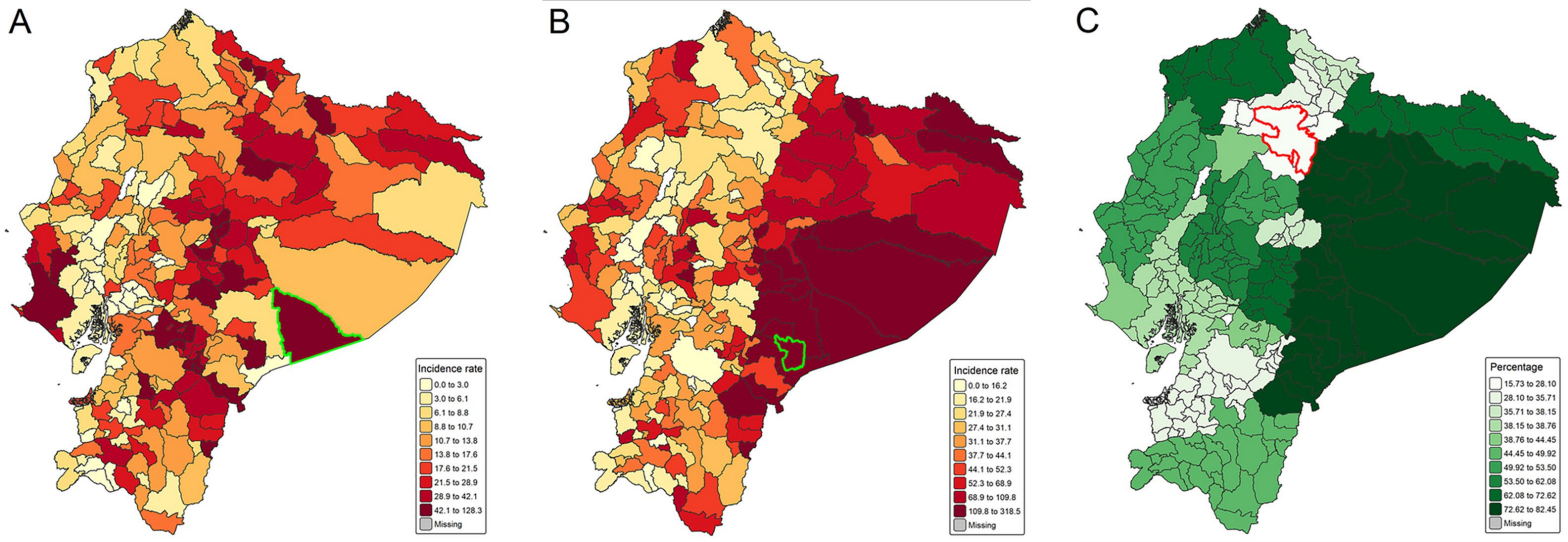
Spatial distribution of undernutrition, intestinal parasitic infections, and multidimensional poverty at the municipal level in Ecuador, 2021. **(A)** Municipal incidence of undernutrition. The green border indicates the municipality with the highest incidence: Taisha. **(B)** Municipal incidence of intestinal parasitic infections. The green border indicates the municipality with the highest incidence: Logroño. Both rates are expressed per 1,000 children-year. **(C)** Proportion of households experiencing multidimensional poverty. The red border indicates Quito municipality, which reported the lowest proportion. Data in all panels are classified using natural breaks (Jenks method) with 10 categories (37).

The national incidence of undernutrition was 14.57 cases per 1,000 children-year (95% confidence interval (95% CI): 14.38–14.75). The Amazon region showed the highest incidence rate per 1,000 children-year (24.15, 95% CI: 23.26–25.06), followed by the Highlands (18.94, 95% CI: 18.62–19.26) and Coast (9.58, 95% CI: 9.36–9.80). The highest undernutrition incidence rate was observed in Taisha (Amazon region), recording 128.30 cases per 1,000 children-year (95% CI: 117.84–139.44) (Figure 1A).

### 3.2 Intestinal parasitic infection

The median age of intestinal parasitic infection cases was 3 years (IQR: 2–3), and there was no significant difference between male and female children (Table 2). Sex distribution was balanced: female children accounted for 50.30% of cases and male children for 49.70% (Table 2). The vast majority of cases (93.98%) were registered as “unspecified helminthiasis,” precluding further taxonomic identification for most entries. Among the cases with an identified pathogen, the most frequent were nematodes of the family Oxyuridea (*Enterobious vermiculari*s) (2.63%), followed by protozoa of the family Entamoebidae (*Entamoeba histolytica*) (~1.5%) and Hexamitidae family (*G. duodenalis*) (1.04%) (Table 2).

**Table 2:** Characterization of cases of intestinal parasitic infections in Ecuadorian children under five years, 2021.

	Overall	Female	Male	Statistical test
	57,203 (100)	28,748 (50.30)	28,455 (49.70)	
Age, median (IQR)	3 (2–3)	3 (2–3)	3 (2–3)	Wilcoxon, *p* = 0.964
Taxonomy				
Amoebozoa*-Evosea-Mastigamoebidae-Entamoebidae, *n* (%)	856 (1.50)	409 (1.42)	447 (1.57)	Chisq. (10 df) = 47.24, *p* < 0.001
Unspecified helminthiasis, *n* (%)	53,759 (93.98)	26,936 (93.70)	26,823 (94.26)	
Matamonada*-Fornicata-Diplomonadida-Hexamitidae, *n* (%)	595 (1.04)	270 (0.94)	325 (1.14)	
Metazoa-Platyhelminthes-Ciclophyllidea-Hymenolepididae, *n* (%)	5 (0.00)	2 (0.01)	3 (0.01)	
Metazoa-Platyhelminthes-Ciclophyllidea-Taeniidae, *n* (%)	12 (0.02)	5 (0.02)	7 (0.02)	
Metazoa-Nematoda-Rhabditida-Ancylostomatidae, *n* (%)	25 (0.04)	12 (0.04)	13 (0.05)	
Metazoa-Nematoda-Rhabditida-Ascarididae, *n* (%)	395 (0.69)	224 (0.78)	171 (0.60)	
Metazoa-Nematoda-Rhabditida-Oxyuridea, *n* (%)	1,503 (2.63)	863 (3.00)	640 (2.25)	
Metazoa-Nematoda-Rhabditida-Strongyloididae, *n* (%)	21 (0.04)	9 (0.03)	12 (0.04)	
Metazoa-Nematoda-Rhabditida-Toxocaridae, *n* (%)	20 (0.04)	11 (0.04)	9 (0.03)	
Metazoa-Nematoda-Trichinellida-Trichuridae, *n* (%)	12 (0.02)	7 (0.02)	5 (0.02)	

Case data were obtained from the 2021 national outpatient‑consultation registry of the Ministerio de Salud Pública del Ecuador (see Methods). Cases were classified according to their assigned ICD‑10 codes (see Methods and Supplementary material 2) and then grouped following a phylogenetic hierarchy: Kingdom → Phylum → Order → Family. IQR, interquartile range; n, number; %, percentage; df, degrees of freedom; *, no phylum has been formally assigned to these organisms.

The national incidence of intestinal parasitic infection was 34.07 cases per 1,000 children-year (95% CI: 33.82–34.35). The Amazon region showed the highest incidence rate per 1,000 children-year (100.88, 95% IC: 98.96–102.84), followed by Coast (29.89, 95% CI: 29.52–30.27) and Highlands (28.73, 95% CI: 28.32–29.15). The highest incidence rate of intestinal parasitic infections was observed in the municipality Logroño (Amazon region), recording 318.53 cases per 1,000 children-year (95% CI: 285.42–354.42) (Figure 1B).

### 3.3 Proportion of multidimensional poverty

Nationally, the median of multidimensional poverty was 44.45% (IQR: 38.04–56.51%). Municipalities surrounding Quito, Ecuador’s capital, exhibited the lowest level of multidimensional poverty (15.74%), whereas Taisha and surrounding areas in the Amazon region recorded the highest (82.40%) (Figure 1C). Regional medians of the proportion of multidimensional poverty differed significantly. The Amazon region demonstrated the highest values (75.95%, IQR: 63.01–82.44%), followed by the Coast (45.93%, IQR: 38.15–49.92%) and Highlands (38.76%, IQR: 35.05–53.17%) (Kruskal-Wallis test, *H* = 72.5, *p* < 0.001; all pairwise comparisons result significant: *p* < 0.001).

### 3.4 Spatial analysis

The spatial autocorrelation analysis revealed significant clustering patterns for undernutrition (Global Moran’s Index = 0.18, *Z* = 4.90, *p* < 0.001), intestinal parasitic infections (Global Moran’s Index = 0.48, *Z* = 12.30, *p* < 0.001), and multidimensional poverty (Global Moran’s Index = 0.69, *Z* = 16.20, *p* < 0.001). Hot spots of undernutrition were predominantly observed in the highlands of Ecuador, particularly in the central and northeastern regions, with additional clusters noted in the northern Amazon (Figure 1A). Intestinal parasitic infections were widespread across the entire Amazon region, with hot spots also appearing along the Coast (Figure 1B). Multidimensional poverty was mainly concentrated in the Amazon, central highlands, and northern coastal areas (Figure 1C).

**Figure 2:**
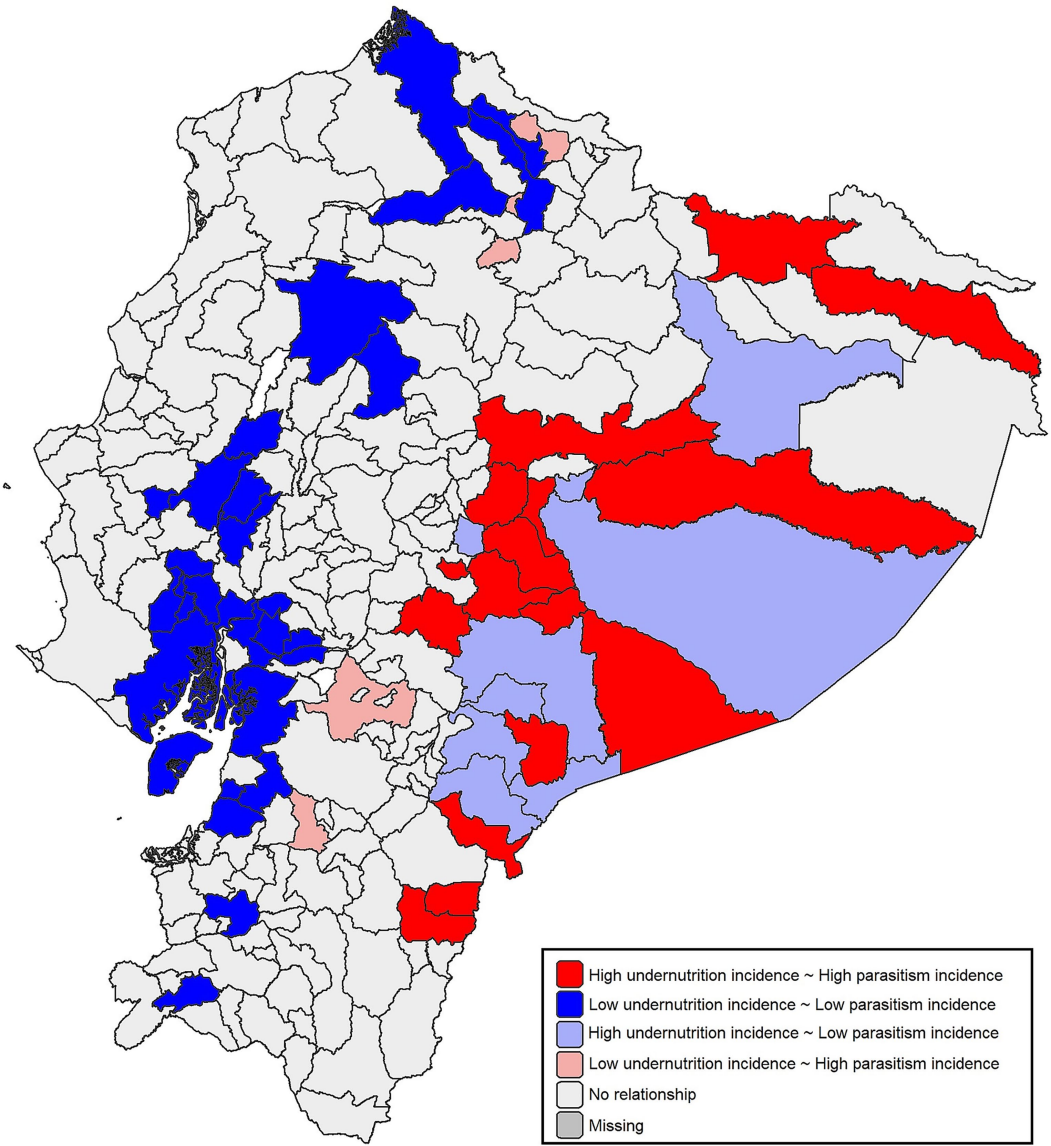
Bivariate spatial clusters of the co‑occurrence of undernutrition and intestinal parasitic infections in Ecuadorian children under 5 years (2021). Municipalities are classified according to the joint spatial distribution of the two incidence rates.

A significant bivariate spatial correlation was found between undernutrition and intestinal parasitic infections (Bivariate Moran’s Index = 0.19, *Z* = 6.04, *p* < 0.001). The analysis identified clusters of municipalities where high incidence rates of both undernutrition and intestinal parasitic infections coexisted (Figure 2). High-high incidence clusters concentrated in the Amazonian provinces of Morona Santiago, Sucumbios, Pastaza, Zamora Chinchipe, and Napo, but were also observed in the Highlands (i.e., Chimborazo, Tungurahua). In contrast, low-low incidence clusters were predominantly located in the Coast, particularly in the province of Guayas, as well as scattered across areas in the Highlands.

Municipalities identified as high-high clusters showed higher multidimensional poverty values compared to low-low clusters. The median multidimensional poverty in high-high municipalities was 72.62% (IQR: 62.08–82.44%), whereas in low-low municipalities it was 38.15% (IQR: 38.15–38.93%) (Wilcoxon rank-sum test, *p* < 0.001). A list of municipalities classified under high-high and low-low clusters is provided in Supplementary material 3.

## 4 Discussion

This study investigated the relationship between multidimensional poverty and the co-occurrence of undernutrition and intestinal parasitic infections among Ecuadorian children under the age of five. Our geospatial analysis revealed that these two conditions cluster within areas experiencing socioeconomic deprivation, particularly in the Amazon region. The pronounced contrast in poverty levels between the high-high incidence clusters and the low-low incidence clusters suggests a strong association between multidimensional poverty and this dual health burden. This pattern is consistent with a syndemic process, in which socioeconomic deprivation creates conditions that foster a vicious cycle between intestinal parasitic infections and undernutrition, thereby exacerbating the overall health burden.

At the individual level, the simultaneous occurrence of both conditions likely results from bidirectional mechanisms that create a synergistic loop (20, 21). Parasites can lower dietary intake, utilize host amino-acids and induce diarrhoea; these effects damage the intestinal mucosa, increase permeability (environmental enteric dysfunction) and trigger systemic inflammation that blunts growth-hormone signaling (20). Simultaneously, undernutrition weakens both innate and adaptive immunity, alters gut-microbiota composition and compromises barrier integrity, thereby increasing susceptibility to parasite colonization, prolonging infection and raising pathogen loads (21). While these biological interactions are crucial at the individual level, our findings indicate that social and structural determinants may underlie this double burden.

In particular, we found that high-high incidence clusters were located in the country’s most deprived areas, as identified by the multidimensional poverty index, whereas low-low clusters occurred in more affluent ones. Within this context, deprivation is defined as the condition in which one-third or more of fundamental needs remain unmet, indicating a systemic failure to guarantee inhabitants’ rights to “*Buen Vivir*” (collective well-being) (22). Moreover, these rights to education, work, social security, health, safe water, nutritious food, a healthy environment, adequate habitat and decent housing are deeply interconnected. Hence, deprivation in any one domain perpetuates challenges in the others, creating a cyclical dynamic that drives the persistence of this double burden.

For example, lack of access to education limits employment opportunities, undermining economic stability (23). As a consequence, families struggle to obtain the nutritious food and health-care necessary for children’s growth, thereby increasing their vulnerability to nutritional deficiencies and infections (9, 24). Child labour is another concern, as it deprives children of schooling and exposes them to unsafe, unhealthy conditions that raise the risk of physical and mental harm (25). Furthermore, lack of access to clean water, sanitation infrastructure, adequate housing and a healthy environment facilitates the transmission of infectious diseases and impedes reliable access to safe, nutritious food. Importantly, our data show that these interconnected deprivations are not evenly distributed. They are concentrated in specific geographical areas, as reflected in the high multidimensional poverty values for Amazonian and Highland provinces (Figure 1C).

Our spatial analysis revealed that high-high clusters are concentrated in regions characterized by intensive extractive economies, specifically oil extraction in the Amazon and large-scale agriculture in the Highlands, which also exhibit the highest levels of multidimensional poverty. This geographic overlap positions extractivism as a plausible, though not definitively proven, structural driver of the observed patterns of multidimensional poverty and its embodied health burden. Influenced by historical and economic forces, an extractive model may generate the conditions for a syndemic process likely through two primary pathways: Nutritional disruption and parasite-transmission amplification (26, 27).

Extractivism systematically undermines nutritional health by displacing traditional food systems. This occurs through the direct loss of hunting grounds, the contamination of soil and waterways, and a forced shift toward a monetized economy, which together dismantle the socio-ecological infrastructure necessary for food sovereignty. Concurrently, the model directly facilitates parasitic transmission by creating and amplifying environmental foci. Excavation pits and pooled water from mining and drilling become breeding grounds for vectors, while the construction of access roads and camps disrupts ecosystems, concentrates human waste, and introduces novel pathogens to previously isolated populations (27–32).

Importantly, the interpretation of our findings must be guided by an understanding of the limitations inherent to ecological designs, principally the ecological fallacy (33). This principle cautions that associations observed at the group level, such as the association between municipal multidimensional poverty and disease clustering, cannot be assumed to hold at the individual level. Our results robustly identify geographic hotspots where a syndemic process is likely occurring at the population scale, but we are unable to identify which specific individuals within those areas are affected, nor can elucidate individual-level mechanisms driving these associations. Therefore, our findings highlight the necessity for complementary methodologies to study them.

### 4.1 Strengths and limitations

This study has several strengths. First, its national scope provides a comprehensive overview of the co-occurrence of undernutrition and intestinal parasitic infections in children under the age of five, allowing for a comparative analysis of regional disparities. Second, the application of geospatial analysis identified clusters of co-occurrence, moving beyond simple mapping to objectively delineate the geographical patterns of the double burden. Finally, the application of empirical Bayesian smoothing was crucial for enhancing the reliability of our incidence estimates, particularly in municipalities with small populations. This technique stabilizes rates by borrowing strength from neighboring municipalities, providing more robust values for the spatial analysis.

However, several limitations must be acknowledged. First, our reliance on routine administrative data may lead to an underestimation of the true disease burden due to underascertainment and diagnostic misclassification (34). Underascertainment—the failure to detect true cases—is likely exacerbated in deprived areas with limited healthcare access. Misclassification, conversely, stems from the limited diagnostic capacity in under-resourced settings, where the use of low-sensitivity techniques can result in false negatives. For our spatial analysis, this measurement error increases the risk of failing to identify all true high-high clusters. Consequently, the clusters we have identified should be interpreted as the minimum detectable geographic areas of this double burden, with the actual burden likely being more severe and widespread.

Additionally, the spatial analysis excluded the Galápagos Islands, three mainland municipalities due to jurisdictional disputes and some cases due to missing information on place of residence. The exclusion of the Galápagos was methodologically necessary, as its extreme geographical distance from the continental landmass violates the fundamental spatial principle of contiguity, preventing meaningful neighborhood analysis. While the exclusion of the three municipalities with disputed borders or cases with missing data ensures the analytical integrity of the spatial clusters, it means our national overview is not based on a complete census of all 221 municipalities and cases. However, given that these exclusions represent a very small fraction of the total under-five population, they are unlikely to alter the overall geographic patterns or conclusions.

Another constraint was the lack of taxonomic specificity in parasite diagnoses, with 94% of them recorded as “unspecified helminthiasis.” While this precludes insights into the specific biological pathways of the co-occurrence, it does not invalidate the primary finding of a spatially clustered disease burden. For the purpose of identifying areas where systemic failures, such as multidimensional poverty, manifest as a high burden of clinically apparent undernutrition and intestinal parasitic infections, this aggregated measure remains a valid indicator. Moreover, the high incidence of unspecified helminthiasis is itself a meaningful finding, reflecting the reality of under-resourced primary care where diagnostic capacity is limited and treatment is often syndromic (35). The reliance on direct saline examination alone, without complementary techniques such as Lugol’s staining, fecal concentration, flotation techniques, sedimentation, or molecular methods, markedly reduces the capacity of parasite detection and identification (35, 36). As a consequence, the true burden of parasitic infections is likely underestimated, and the identified geographic patterns likely are a conservative representation of the problem.

## 5 Conclusion

This geospatial analysis documents a concentration of child undernutrition and intestinal parasitic infections in Ecuador’s most impoverished municipalities, especially in the Amazon. The spatial co-location of this dual burden with regions of high multidimensional poverty supports the notion of a syndemic process. Because the study is ecological, the findings identify crucial area-level patterns for public-health planning; however, caution is needed when extrapolating these results to individuals, and further research is required. The results show how systemic failures in social and environmental justice can become embodied as population-level health patterns. From a critical perspective, this highlights the need for structural interventions that guarantee the rights to *Buen Vivir*—including food sovereignty, territorial integrity, and a healthy environment—as fundamental prerequisites for health equity.

## Data Availability

The original contributions presented in the study are included in the article/Supplementary material, further inquiries can be directed to the corresponding author.
