# Supplementary material for: Multidimensional poverty and the co-occurrence of undernutrition and intestinal parasitic infections in Ecuadorian infants: a geospatial analysis
Source: Front Public Health. 2025 Nov 19;13:1668303. doi: 10.3389/fpubh.2025.1668303 (PMC12672338; doi:10.3389/fpubh.2025.1668303)
Supplement: Supplementary file 1 [file Data_Sheet_1.pdf]

Supplementary material 1. Regional distribution of Ecuador.

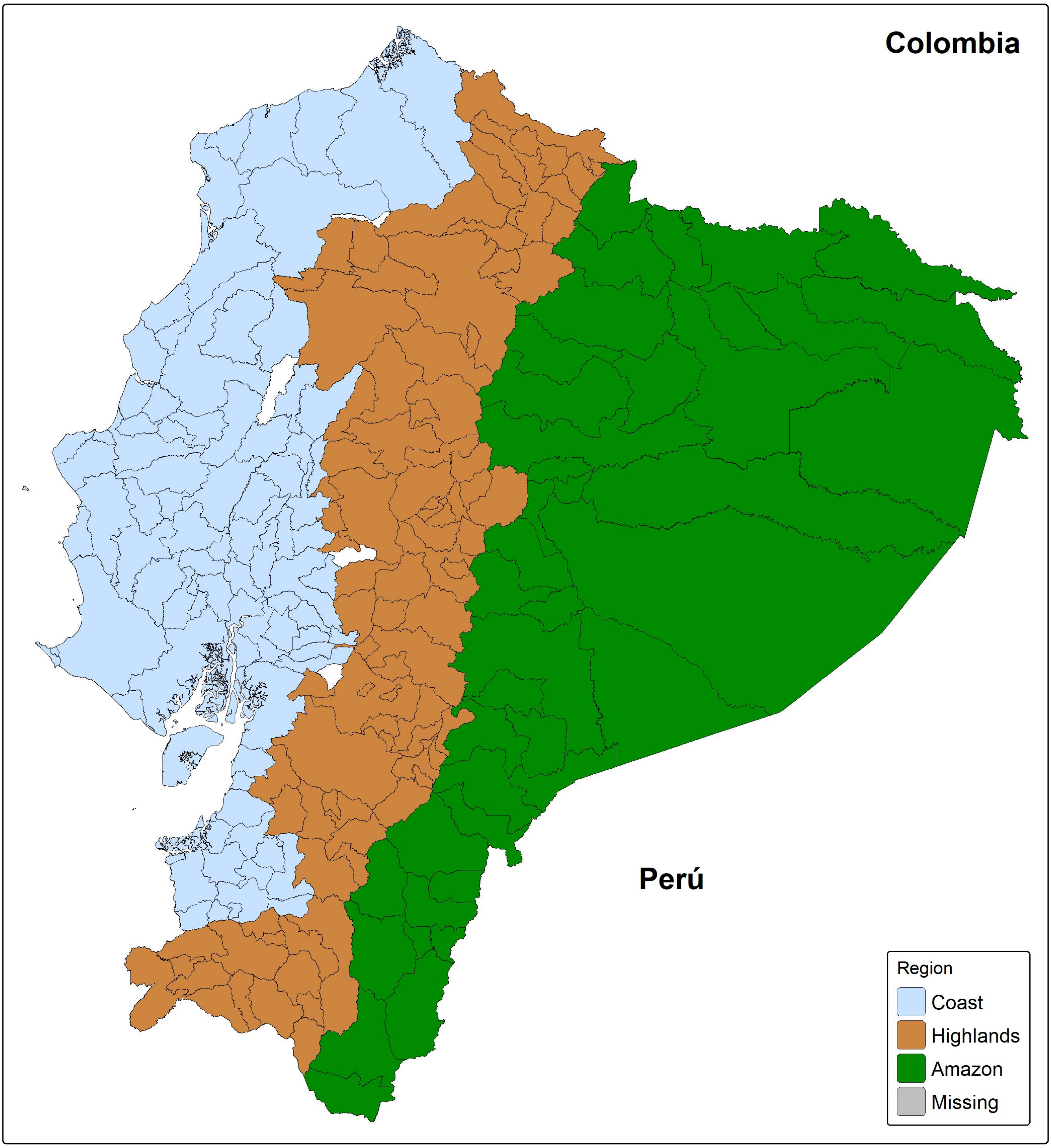

The country's regions are shown in the lower right corner. The geographic boundaries are also shown: Colombia to the north and east, and Peru to the south and east.
