# Supplementary material for: Multidimensional poverty and the co-occurrence of undernutrition and intestinal parasitic infections in Ecuadorian infants: a geospatial analysis
Source: Front Public Health. 2025 Nov 19;13:1668303. doi: 10.3389/fpubh.2025.1668303 (PMC12672338; doi:10.3389/fpubh.2025.1668303)
Supplement: Supplementary file 2 [file Data_Sheet_2.docx]

**Supplementary Material 2.** ICD-10 codes used to identify cases of intestinal parasitic infections.

| **Code** | **Categorisation** | **ICD-10 definition** | **Pathogen** |
| --- | --- | --- | --- |
| A060 | Amoebozoa*-Evosea-Mastigamoebidae-Entamoebidae | Acute amoebic dysentery | *E. histolytica* |
| A061 | Amoebozoa*-Evosea-Mastigamoebidae-Entamoebidae | Chronic intestinal amoebiasis | *E. histolytica* |
| A062 | Amoebozoa*-Evosea-Mastigamoebidae-Entamoebidae | Non-dysenteric amoebic colitis | *E. histolytica* |
| A063 | Amoebozoa*-Evosea-Mastigamoebidae-Entamoebidae | Intestinal amoeboma | *E. histolytica* |
| A064 | Amoebozoa*-Evosea-Mastigamoebidae-Entamoebidae | Amoebic liver abscess | *E. histolytica* |
| A071 | Matamonada*-Fornicata-Diplomonadida-Hexamitidae | Giardiasis [lambliasis] | *G. duodenalis* |
| B681 | Metazoa-Platyhelminthes-Ciclophyllidea-Taeniidae | Infection due to Taenia saginata | *Taennia saginata* |
| B689 | Metazoa-Platyhelminthes-Ciclophyllidea-Taeniidae | Unspecified taeniasis | Unspecified taeniasis |
| B710 | Metazoa-Platyhelminthes-Ciclophyllidea-Hymenolepididae | Himenolepiasis | *Hymenolepis nana* |
| B760 | Metazoa-Nematoda-Rhabditida-Ancylostomatidae | Ancylostomiasis | Anquilostomiasis |
| B761 | Metazoa-Nematoda-Rhabditida-Ancylostomatidae | Necatoriasis | *N. americanus* |
| B769 | Metazoa-Nematoda-Rhabditida-Ancylostomatidae | Ancylostomiasis, unspecified | Anquilostomiasis |
| B770 | Metazoa-Nematoda-Rhabditida-Ascarididae | Ascariasis with intestinal complications | *A. lumbricoides* |
| B778 | Metazoa-Nematoda-Rhabditida-Ascarididae | Ascariasis with other complications | *A. lumbricoides* |
| B779 | Metazoa-Nematoda-Rhabditida-Ascarididae | Ascariasis, unspecified | *A. lumbricoides* |
| B780 | Metazoa-Nematoda-Rhabditida-Strongyloidiasis | Intestinal strongyloidiasis | *S. stercoralis* |
| B781 | Metazoa-Nematoda-Rhabditida-Strongyloidiasis | Cutaneous strongyloidiasis | *S. stercoralis* |
| B789 | Metazoa-Nematoda-Rhabditida-Strongyloididae | Strongyloidiasis, unspecified | *S. stercoralis* |
| B79X | Metazoa-Nematoda-Trichinellida-Trichurida | Trichuriasis | *T. trichiura* |
| B80X | Metazoa-Nematoda-Rhabditida-Oxyuridea | Enterobiasis | *E. vermicularis* |
| B814 | Unspecified helminthiasis | Mixed intestinal helminthiases | Helminthiasis, unspecified |
| B818 | Unspecified helminthiasis | Other specified intestinal helminthiases | Helminthiasis, unspecified |
| B820 | Unspecified helminthiasis | Intestinal helminthiasis, unspecified | Helminthiasis, unspecified |
| B829 | Unspecified helminthiasis | Intestinal parasitism, unspecified | Helminthiasis, unspecified |
| B830 | Metazoa-Nematoda-Rhabditida-Toxocaridae | Visceral larva migrans | *Toxocara* |
| B838 | Unspecified helminthiasis | Other specified helminthiases | Helminthiasis, unspecified |
| B839 | Unspecified helminthiasis | Helminthiasis, unspecified | Helminthiasis, unspecified |

The table includes the classification category, official ICD-10 diagnostic term (ICD-10 definition), and corresponding pathogen. Codes correspond to protozoal (e.g., *E. histolytica,* *G. duodenalis*) and helminthic infections (e.g., *A. lumbricoides*, *S. stercoralis*, *T. trichiura*), with taxonomic categorization performed up to the family or genus level where applicable. Records labeled as "Helminthiasis, unspecified" were retained in the analysis under the category of non-specified parasitosis.
