# Supplementary material for: Multidimensional poverty and the co-occurrence of undernutrition and intestinal parasitic infections in Ecuadorian infants: a geospatial analysis
Source: Front Public Health. 2025 Nov 19;13:1668303. doi: 10.3389/fpubh.2025.1668303 (PMC12672338; doi:10.3389/fpubh.2025.1668303)
Supplement: Supplementary file 3 [file Data_Sheet_3.docx]

| **Supplementary material 3. Incidence rates calculated for MUI and MPI. Municipal PMP. Ecuador 2021.** Clusters of high-high and low-low incidence were located using bivariate Moran’s Index (see Methods section). *Abbreviations: und_cas, cases of undernutrition; MUI, Municipal Undernutrition Incidence; MUI_ciL, lower 95% confidence interval of MUI; MUI_ciU, upper 95% confidence interval of MUI; MUI_bay, Bayesian correction of MUI; ipi_cas, cases of intestinal parasitic infection; MPI, Municipal Intestinal Parasitosis Incidence; MPI_ciL, lower 95% confidence interval of MPI; MPI_ciU, upper 95% confidence interval of MPI; MPI_bay, Bayesian correction of MPI; MP, multidimensional poverty; H-H, High MUI - High MPI; L-L, Low MUI - Low MPI; H-L, High MUI - Low MPI; L-H, Low MUI - High MPI.* | | | | | | | | | | | | | | |
| --- | --- | --- | --- | --- | --- | --- | --- | --- | --- | --- | --- | --- | --- | --- |
|  |  |  |  |  |  |  |  |  |  |  |  |  |  |  |
| **Municipality** | **Region** | **Province** | **und_cas** | **MUI** | **MUI_ciL** | **MUI_ciU** | **MUI_bay** | **ipi_cas** | **MPI** | **MPI_ciL** | **MPI_ciU** | **MPI_bay** | **MP** | **Cluster** |
| GUARANDA | Highlands | BOLIVAR | 159 | 13.62 | 11.58 | 15.9 | 0.01 | 284 | 24.32 | 21.57 | 27.32 | 0.02 | 53.5 | - |
| CHILLANES | Highlands | BOLIVAR | 57 | 28.67 | 21.72 | 37.15 | 0.03 | 74 | 37.22 | 29.23 | 46.73 | 0.04 | 53.5 | - |
| CHIMBO | Highlands | BOLIVAR | 0 | 0 | 0 | 2.18 | 0 | 0 | 0 | 0 | 2.18 | 0 | 53.5 | - |
| ECHEANDIA | Highlands | BOLIVAR | 28 | 23.12 | 15.36 | 33.42 | 0.02 | 139 | 114.78 | 96.49 | 135.53 | 0.11 | 53.5 | - |
| SAN MIGUEL | Highlands | BOLIVAR | 28 | 9.77 | 6.49 | 14.12 | 0.01 | 94 | 32.79 | 26.5 | 40.12 | 0.03 | 53.5 | - |
| CALUMA | Highlands | BOLIVAR | 2 | 1.36 | 0.16 | 4.9 | 0 | 68 | 46.1 | 35.8 | 58.44 | 0.05 | 53.5 | - |
| LAS NAVES | Highlands | BOLIVAR | 5 | 7.47 | 2.43 | 17.44 | 0.01 | 15 | 22.42 | 12.55 | 36.98 | 0.02 | 53.5 | - |
| TULCAN | Highlands | CARCHI | 276 | 28.84 | 25.54 | 32.45 | 0.03 | 260 | 27.17 | 23.97 | 30.68 | 0.03 | 35.71 | - |
| BOLIVAR | Highlands | CARCHI | 0 | 0 | 0 | 2.34 | 0 | 0 | 0 | 0 | 2.34 | 0 | 35.71 | - |
| ESPEJO | Highlands | CARCHI | 65 | 47.9 | 36.97 | 61.05 | 0.05 | 18 | 13.26 | 7.86 | 20.96 | 0.01 | 35.71 | L-H |
| MIRA | Highlands | CARCHI | 13 | 11.42 | 6.08 | 19.53 | 0.01 | 37 | 32.51 | 22.89 | 44.82 | 0.03 | 35.71 | L-L |
| MONTUFAR | Highlands | CARCHI | 110 | 33.21 | 27.3 | 40.03 | 0.03 | 83 | 25.06 | 19.96 | 31.07 | 0.03 | 35.71 | - |
| SAN PEDRO DE HUACA | Highlands | CARCHI | 11 | 14.53 | 7.25 | 26 | 0.01 | 29 | 38.31 | 25.66 | 55.02 | 0.04 | 35.71 | - |
| LATACUNGA | Highlands | COTOPAXI | 466 | 24.39 | 22.23 | 26.71 | 0.02 | 716 | 37.48 | 34.79 | 40.33 | 0.04 | 53.18 | - |
| LA MANA | Highlands | COTOPAXI | 28 | 5.21 | 3.46 | 7.52 | 0.01 | 221 | 41.09 | 35.85 | 46.87 | 0.04 | 53.18 | - |
| PANGUA | Highlands | COTOPAXI | 50 | 17.6 | 13.06 | 23.2 | 0.02 | 224 | 78.85 | 68.86 | 89.87 | 0.08 | 53.18 | - |
| PUJILI | Highlands | COTOPAXI | 359 | 40.18 | 36.13 | 44.56 | 0.04 | 262 | 29.33 | 25.88 | 33.1 | 0.03 | 53.18 | - |
| SALCEDO | Highlands | COTOPAXI | 230 | 37.11 | 32.47 | 42.23 | 0.04 | 400 | 64.55 | 58.38 | 71.19 | 0.06 | 53.18 | - |
| SAQUISILI | Highlands | COTOPAXI | 80 | 24.02 | 19.05 | 29.9 | 0.02 | 45 | 13.51 | 9.86 | 18.08 | 0.01 | 53.18 | - |
| SIGCHOS | Highlands | COTOPAXI | 28 | 8.77 | 5.83 | 12.67 | 0.01 | 70 | 21.92 | 17.09 | 27.7 | 0.02 | 53.18 | L-L |
| RIOBAMBA | Highlands | CHIMBORAZO | 904 | 39.08 | 36.58 | 41.71 | 0.04 | 1041 | 45 | 42.31 | 47.82 | 0.04 | 62.08 | - |
| ALAUSI | Highlands | CHIMBORAZO | 98 | 16.03 | 13.01 | 19.53 | 0.02 | 147 | 24.04 | 20.31 | 28.26 | 0.02 | 62.08 | - |
| COLTA | Highlands | CHIMBORAZO | 126 | 28.21 | 23.5 | 33.58 | 0.03 | 238 | 53.28 | 46.73 | 60.5 | 0.05 | 62.08 | - |
| CHAMBO | Highlands | CHIMBORAZO | 35 | 26.06 | 18.15 | 36.24 | 0.03 | 95 | 70.74 | 57.23 | 86.47 | 0.07 | 62.08 | H-H |
| CHUNCHI | Highlands | CHIMBORAZO | 14 | 9.46 | 5.17 | 15.87 | 0.01 | 56 | 37.84 | 28.58 | 49.14 | 0.04 | 62.08 | - |
| GUAMOTE | Highlands | CHIMBORAZO | 371 | 54.55 | 49.14 | 60.39 | 0.05 | 147 | 21.61 | 18.26 | 25.4 | 0.02 | 62.08 | H-H |
| GUANO | Highlands | CHIMBORAZO | 230 | 46.38 | 40.58 | 52.78 | 0.05 | 203 | 40.94 | 35.5 | 46.97 | 0.04 | 62.08 | - |
| PALLATANGA | Highlands | CHIMBORAZO | 25 | 16.67 | 10.79 | 24.6 | 0.02 | 41 | 27.33 | 19.61 | 37.08 | 0.03 | 62.08 | - |
| PENIPE | Highlands | CHIMBORAZO | 12 | 17.67 | 9.13 | 30.87 | 0.02 | 78 | 114.87 | 90.8 | 143.37 | 0.11 | 62.08 | H-L |

| CUMANDA | Highlands | CHIMBORAZO | 13 | 7.64 | 4.07 | 13.06 | 0.01 | 70 | 41.13 | 32.06 | 51.96 | 0.04 | 62.08 | - |
| --- | --- | --- | --- | --- | --- | --- | --- | --- | --- | --- | --- | --- | --- | --- |
| ESMERALDAS | Coast | ESMERALDAS | 165 | 6.98 | 5.96 | 8.14 | 0.01 | 1055 | 44.66 | 42.01 | 47.44 | 0.04 | 63.16 | - |
| ELOY ALFARO | Coast | ESMERALDAS | 54 | 8.77 | 6.59 | 11.45 | 0.01 | 133 | 21.61 | 18.1 | 25.61 | 0.02 | 63.16 | - |
| MUISNE | Coast | ESMERALDAS | 24 | 5.8 | 3.71 | 8.63 | 0.01 | 123 | 29.71 | 24.69 | 35.45 | 0.03 | 63.16 | - |
| QUININDE | Coast | ESMERALDAS | 349 | 19.97 | 17.93 | 22.18 | 0.02 | 772 | 44.17 | 41.11 | 47.4 | 0.04 | 63.16 | - |
| SAN LORENZO | Coast | ESMERALDAS | 56 | 7.96 | 6.01 | 10.33 | 0.01 | 292 | 41.49 | 36.87 | 46.53 | 0.04 | 63.16 | L-L |
| ATACAMES | Coast | ESMERALDAS | 117 | 19.71 | 16.3 | 23.62 | 0.02 | 169 | 28.47 | 24.34 | 33.1 | 0.03 | 63.16 | - |
| RIOVERDE | Coast | ESMERALDAS | 31 | 7.51 | 5.1 | 10.66 | 0.01 | 358 | 86.7 | 77.95 | 96.17 | 0.09 | 63.16 | - |
| IBARRA | Highlands | IMBABURA | 277 | 14.49 | 12.83 | 16.3 | 0.01 | 478 | 25 | 22.81 | 27.34 | 0.03 | 35.06 | L-L |
| ANTONIO ANTE | Highlands | IMBABURA | 238 | 48.51 | 42.54 | 55.08 | 0.05 | 255 | 51.98 | 45.79 | 58.76 | 0.05 | 35.06 | L-H |
| COTACACHI | Highlands | IMBABURA | 47 | 9.29 | 6.82 | 12.35 | 0.01 | 123 | 24.3 | 20.2 | 29 | 0.02 | 35.06 | L-L |
| OTAVALO | Highlands | IMBABURA | 199 | 15.64 | 13.54 | 17.97 | 0.02 | 217 | 17.06 | 14.86 | 19.48 | 0.02 | 35.06 | - |
| PIMAMPIRO | Highlands | IMBABURA | 33 | 24.87 | 17.12 | 34.92 | 0.02 | 82 | 61.79 | 49.15 | 76.7 | 0.06 | 35.06 | - |
| SAN MIGUEL DE URCUQUI | Highlands | IMBABURA | 32 | 17.68 | 12.09 | 24.96 | 0.02 | 26 | 14.36 | 9.38 | 21.05 | 0.01 | 35.06 | - |
| TENA | Amazon | NAPO | 263 | 27.93 | 24.66 | 31.52 | 0.03 | 741 | 78.7 | 73.13 | 84.57 | 0.08 | 75.96 | H-H |
| ARCHIDONA | Amazon | NAPO | 73 | 18.04 | 14.14 | 22.69 | 0.02 | 240 | 59.32 | 52.05 | 67.32 | 0.06 | 75.96 | - |
| EL CHACO | Amazon | NAPO | 30 | 29.79 | 20.1 | 42.53 | 0.03 | 91 | 90.37 | 72.76 | 110.95 | 0.09 | 75.96 | - |
| QUIJOS | Amazon | NAPO | 36 | 51.28 | 35.92 | 71 | 0.05 | 77 | 109.69 | 86.56 | 137.09 | 0.11 | 75.96 | - |
| CARLOS JULIO AROSEMENA TOLA | Amazon | NAPO | 4 | 7.5 | 2.04 | 19.21 | 0.01 | 23 | 43.15 | 27.35 | 64.75 | 0.04 | 75.96 | - |
| PASTAZA | Amazon | PASTAZA | 83 | 9.22 | 7.34 | 11.43 | 0.01 | 992 | 110.21 | 103.46 | 117.29 | 0.11 | 72.62 | H-L |
| MERA | Amazon | PASTAZA | 46 | 29.53 | 21.62 | 39.38 | 0.03 | 162 | 103.98 | 88.58 | 121.28 | 0.1 | 72.62 | H-H |
| SANTA CLARA | Amazon | PASTAZA | 3 | 6.3 | 1.3 | 18.42 | 0.01 | 67 | 140.76 | 109.08 | 178.76 | 0.13 | 72.62 | H-L |
| ARAJUNO | Amazon | PASTAZA | 28 | 21.02 | 13.97 | 30.38 | 0.02 | 205 | 153.9 | 133.56 | 176.48 | 0.15 | 72.62 | H-H |
| CAYAMBE | Highlands | PICHINCHA | 465 | 41.93 | 38.2 | 45.92 | 0.04 | 339 | 30.57 | 27.4 | 34 | 0.03 | 15.73 | - |
| MEJIA | Highlands | PICHINCHA | 194 | 20.52 | 17.73 | 23.62 | 0.02 | 278 | 29.41 | 26.05 | 33.07 | 0.03 | 15.73 | - |
| PEDRO MONCAYO | Highlands | PICHINCHA | 85 | 19.22 | 15.35 | 23.76 | 0.02 | 114 | 25.77 | 21.26 | 30.96 | 0.03 | 15.73 | L-H |
| RUMIÑAHUI | Highlands | PICHINCHA | 137 | 16.07 | 13.49 | 19 | 0.02 | 198 | 23.23 | 20.1 | 26.7 | 0.02 | 15.73 | - |
| SAN MIGUEL DE LOS BANCOS | Highlands | PICHINCHA | 75 | 31.9 | 25.09 | 39.99 | 0.03 | 76 | 32.33 | 25.47 | 40.46 | 0.03 | 15.73 | - |
| PEDRO VICENTE MALDONADO | Highlands | PICHINCHA | 37 | 19.37 | 13.64 | 26.7 | 0.02 | 59 | 30.89 | 23.51 | 39.85 | 0.03 | 15.73 | - |
| PUERTO QUITO | Highlands | PICHINCHA | 50 | 18.05 | 13.4 | 23.8 | 0.02 | 94 | 33.94 | 27.42 | 41.53 | 0.03 | 15.73 | - |

| AMBATO | Highlands | TUNGURAHUA | 438 | 12.92 | 11.74 | 14.19 | 0.01 | 1099 | 32.42 | 30.53 | 34.4 | 0.03 | 38.05 | - |
| --- | --- | --- | --- | --- | --- | --- | --- | --- | --- | --- | --- | --- | --- | --- |
| BAÑOS DE AGUA SANTA | Highlands | TUNGURAHUA | 75 | 38.03 | 29.91 | 47.67 | 0.04 | 126 | 63.89 | 53.23 | 76.07 | 0.06 | 38.05 | H-H |
| CEVALLOS | Highlands | TUNGURAHUA | 7 | 8.36 | 3.36 | 17.23 | 0.01 | 62 | 74.07 | 56.79 | 94.96 | 0.07 | 38.05 | - |
| MOCHA | Highlands | TUNGURAHUA | 2 | 3.02 | 0.37 | 10.91 | 0 | 33 | 49.85 | 34.31 | 70.01 | 0.05 | 38.05 | - |
| PATATE | Highlands | TUNGURAHUA | 69 | 46.46 | 36.15 | 58.8 | 0.04 | 165 | 111.11 | 94.8 | 129.42 | 0.11 | 38.05 | - |
| QUERO | Highlands | TUNGURAHUA | 55 | 25.93 | 19.53 | 33.75 | 0.03 | 65 | 30.65 | 23.65 | 39.06 | 0.03 | 38.05 | - |
| SAN PEDRO DE PELILEO | Highlands | TUNGURAHUA | 291 | 47.89 | 42.54 | 53.72 | 0.05 | 230 | 37.85 | 33.11 | 43.07 | 0.04 | 38.05 | - |
| SANTIAGO DE PILLARO | Highlands | TUNGURAHUA | 173 | 45.12 | 38.65 | 52.37 | 0.04 | 199 | 51.9 | 44.94 | 59.64 | 0.05 | 38.05 | - |
| TISALEO | Highlands | TUNGURAHUA | 11 | 9.05 | 4.52 | 16.2 | 0.01 | 56 | 46.09 | 34.82 | 59.85 | 0.05 | 38.05 | - |
| LAGO AGRIO | Amazon | SUCUMBIOS | 248 | 20.09 | 17.66 | 22.75 | 0.02 | 1090 | 88.28 | 83.12 | 93.68 | 0.09 | 63.02 | H-H |
| GONZALO PIZARRO | Amazon | SUCUMBIOS | 21 | 16.73 | 10.36 | 25.58 | 0.02 | 99 | 78.88 | 64.11 | 96.04 | 0.08 | 63.02 | - |
| PUTUMAYO | Amazon | SUCUMBIOS | 46 | 28.07 | 20.55 | 37.44 | 0.03 | 217 | 132.4 | 115.37 | 151.23 | 0.13 | 63.02 | - |
| SHUSHUFINDI | Amazon | SUCUMBIOS | 159 | 26.43 | 22.48 | 30.87 | 0.03 | 427 | 70.98 | 64.4 | 78.04 | 0.07 | 63.02 | - |
| SUCUMBIOS | Amazon | SUCUMBIOS | 6 | 14.18 | 5.21 | 30.87 | 0.01 | 28 | 66.19 | 43.99 | 95.67 | 0.06 | 63.02 | - |
| CASCALES | Amazon | SUCUMBIOS | 93 | 52.87 | 42.67 | 64.77 | 0.05 | 245 | 139.28 | 122.39 | 157.86 | 0.14 | 63.02 | - |
| CUYABENO | Amazon | SUCUMBIOS | 40 | 36.2 | 25.86 | 49.29 | 0.03 | 123 | 111.31 | 92.51 | 132.81 | 0.11 | 63.02 | H-H |
| ORELLANA | Amazon | ORELLANA | 170 | 14.96 | 12.8 | 17.39 | 0.01 | 650 | 57.22 | 52.9 | 61.79 | 0.06 | 79.6 | H-L |
| AGUARICO | Amazon | ORELLANA | 5 | 6.07 | 1.97 | 14.16 | 0.01 | 79 | 95.87 | 75.9 | 119.49 | 0.09 | 79.6 | - |
| LA JOYA DE LOS SACHAS | Amazon | ORELLANA | 54 | 10.06 | 7.56 | 13.13 | 0.01 | 235 | 43.79 | 38.37 | 49.76 | 0.04 | 79.6 | - |
| LORETO | Amazon | ORELLANA | 117 | 29.78 | 24.63 | 35.69 | 0.03 | 297 | 75.59 | 67.24 | 84.7 | 0.08 | 79.6 | - |
| SANTO DOMINGO | Highlands | SANTO DOMINGO | 492 | 10.56 | 9.65 | 11.54 | 0.01 | 1230 | 26.41 | 24.95 | 27.93 | 0.03 | 41.27 | L-L |
| QUITO | Highlands | PICHINCHA | 2907 | 12.5 | 12.05 | 12.96 | 0.01 | 5073 | 21.82 | 21.22 | 22.42 | 0.02 | 15.73 | - |
| LA CONCORDIA | Highlands | ESMERALDAS | 142 | 24.29 | 20.46 | 28.62 | 0.02 | 326 | 55.76 | 49.87 | 62.15 | 0.06 | 63.16 | - |
| CUENCA | Highlands | AZUAY | 670 | 12.21 | 11.31 | 13.17 | 0.01 | 684 | 12.47 | 11.55 | 13.44 | 0.01 | 28.1 | - |
| GIRON | Highlands | AZUAY | 13 | 9.11 | 4.85 | 15.58 | 0.01 | 31 | 21.72 | 14.76 | 30.84 | 0.02 | 28.1 | - |
| GUALACEO | Highlands | AZUAY | 225 | 44.44 | 38.82 | 50.64 | 0.04 | 144 | 28.44 | 23.99 | 33.48 | 0.03 | 28.1 | - |
| PAUTE | Highlands | AZUAY | 96 | 32.54 | 26.36 | 39.74 | 0.03 | 167 | 56.61 | 48.35 | 65.88 | 0.06 | 28.1 | - |
| PUCARA | Highlands | AZUAY | 12 | 9.38 | 4.85 | 16.39 | 0.01 | 33 | 25.8 | 17.76 | 36.23 | 0.03 | 28.1 | - |
| SAN FERNANDO | Highlands | AZUAY | 4 | 10.67 | 2.91 | 27.31 | 0.01 | 4 | 10.67 | 2.91 | 27.31 | 0.01 | 28.1 | - |
| SANTA ISABEL | Highlands | AZUAY | 93 | 43.79 | 35.34 | 53.64 | 0.04 | 69 | 32.49 | 25.28 | 41.11 | 0.03 | 28.1 | L-H |

| SIGSIG | Highlands | AZUAY | 115 | 35.7 | 29.48 | 42.86 | 0.04 | 191 | 59.3 | 51.19 | 68.33 | 0.06 | 28.1 | - |
| --- | --- | --- | --- | --- | --- | --- | --- | --- | --- | --- | --- | --- | --- | --- |
| OÑA | Highlands | AZUAY | 7 | 17.07 | 6.86 | 35.18 | 0.02 | 10 | 24.39 | 11.7 | 44.85 | 0.03 | 28.1 | - |
| CHORDELEG | Highlands | AZUAY | 163 | 112.8 | 96.15 | 131.51 | 0.11 | 34 | 23.53 | 16.29 | 32.88 | 0.02 | 28.1 | - |
| EL PAN | Highlands | AZUAY | 10 | 38.02 | 18.23 | 69.93 | 0.03 | 23 | 87.45 | 55.44 | 131.22 | 0.08 | 28.1 | - |
| SEVILLA DE ORO | Highlands | AZUAY | 9 | 13.24 | 6.05 | 25.12 | 0.01 | 30 | 44.12 | 29.77 | 62.98 | 0.04 | 28.1 | - |
| GUACHAPALA | Highlands | AZUAY | 18 | 47.12 | 27.93 | 74.47 | 0.04 | 15 | 39.27 | 21.98 | 64.76 | 0.04 | 28.1 | - |
| CAMILO PONCE ENRIQUEZ | Highlands | AZUAY | 40 | 13.76 | 9.83 | 18.74 | 0.01 | 117 | 40.25 | 33.29 | 48.24 | 0.04 | 28.1 | L-L |
| AZOGUES | Highlands | CAÑAR | 287 | 37.53 | 33.31 | 42.13 | 0.04 | 431 | 56.36 | 51.17 | 61.94 | 0.06 | 38.76 | - |
| BIBLIAN | Highlands | CAÑAR | 140 | 59.47 | 50.03 | 70.18 | 0.06 | 67 | 28.46 | 22.06 | 36.15 | 0.03 | 38.76 | - |
| CAÑAR | Highlands | CAÑAR | 355 | 51.4 | 46.2 | 57.04 | 0.05 | 238 | 34.46 | 30.22 | 39.13 | 0.03 | 38.76 | L-H |
| LA TRONCAL | Highlands | CAÑAR | 109 | 15.16 | 12.45 | 18.28 | 0.02 | 212 | 29.48 | 25.65 | 33.73 | 0.03 | 38.76 | - |
| EL TAMBO | Highlands | CAÑAR | 41 | 36.71 | 26.34 | 49.8 | 0.04 | 18 | 16.11 | 9.55 | 25.47 | 0.02 | 38.76 | - |
| DELEG | Highlands | CAÑAR | 14 | 21.57 | 11.79 | 36.19 | 0.02 | 47 | 72.42 | 53.21 | 96.3 | 0.07 | 38.76 | - |
| SUSCAL | Highlands | CAÑAR | 17 | 30.52 | 17.78 | 48.87 | 0.03 | 17 | 30.52 | 17.78 | 48.87 | 0.03 | 38.76 | - |
| MACHALA | Coast | EL ORO | 241 | 9.36 | 8.22 | 10.62 | 0.01 | 575 | 22.33 | 20.54 | 24.23 | 0.02 | 28.8 | - |
| ATAHUALPA | Coast | EL ORO | 2 | 3.6 | 0.44 | 12.99 | 0 | 10 | 17.99 | 8.62 | 33.08 | 0.02 | 28.8 | - |
| BALSAS | Coast | EL ORO | 5 | 6.01 | 1.95 | 14.02 | 0.01 | 29 | 34.86 | 23.34 | 50.06 | 0.03 | 28.8 | - |
| CHILLA | Coast | EL ORO | 0 | 0 | 0 | 12.94 | 0 | 14 | 49.12 | 26.86 | 82.42 | 0.05 | 28.8 | - |
| EL GUABO | Coast | EL ORO | 58 | 9.28 | 7.04 | 11.99 | 0.01 | 190 | 30.39 | 26.22 | 35.03 | 0.03 | 28.8 | L-L |
| MARCABELI | Coast | EL ORO | 7 | 12.13 | 4.88 | 25 | 0.01 | 11 | 19.06 | 9.52 | 34.11 | 0.02 | 28.8 | - |
| PASAJE | Coast | EL ORO | 69 | 9.09 | 7.07 | 11.5 | 0.01 | 227 | 29.9 | 26.13 | 34.05 | 0.03 | 28.8 | - |
| PIÑAS | Coast | EL ORO | 48 | 18.55 | 13.68 | 24.59 | 0.02 | 141 | 54.48 | 45.86 | 64.25 | 0.05 | 28.8 | L-L |
| PORTOVELO | Coast | EL ORO | 24 | 19.42 | 12.44 | 28.89 | 0.02 | 65 | 52.59 | 40.59 | 67.03 | 0.05 | 28.8 | - |
| SANTA ROSA | Coast | EL ORO | 144 | 20.19 | 17.03 | 23.77 | 0.02 | 222 | 31.12 | 27.16 | 35.5 | 0.03 | 28.8 | - |
| ZARUMA | Coast | EL ORO | 10 | 4.27 | 2.05 | 7.86 | 0 | 92 | 39.3 | 31.68 | 48.2 | 0.04 | 28.8 | - |
| LAS LAJAS | Coast | EL ORO | 0 | 0 | 0 | 8.25 | 0 | 34 | 76.06 | 52.68 | 106.29 | 0.07 | 28.8 | - |
| GUAYAQUIL | Coast | GUAYAS | 1266 | 4.95 | 4.68 | 5.23 | 0 | 4504 | 17.61 | 17.1 | 18.13 | 0.02 | 38.15 | L-L |
| ALFREDO BAQUERIZO MORENO | Coast | GUAYAS | 7 | 2.27 | 0.91 | 4.68 | 0 | 92 | 29.88 | 24.09 | 36.64 | 0.03 | 38.15 | L-L |
| BALAO | Coast | GUAYAS | 2 | 0.71 | 0.09 | 2.56 | 0 | 47 | 16.67 | 12.25 | 22.17 | 0.02 | 38.15 | - |
| BALZAR | Coast | GUAYAS | 14 | 1.95 | 1.07 | 3.28 | 0 | 79 | 11.02 | 8.72 | 13.73 | 0.01 | 38.15 | L-L |
| COLIMES | Coast | GUAYAS | 11 | 3.5 | 1.75 | 6.26 | 0 | 10 | 3.18 | 1.53 | 5.85 | 0 | 38.15 | - |
| DAULE | Coast | GUAYAS | 15 | 1.06 | 0.59 | 1.74 | 0 | 86 | 6.06 | 4.84 | 7.48 | 0.01 | 38.15 | L-L |

| DURAN | Coast | GUAYAS | 65 | 2.42 | 1.87 | 3.09 | 0 | 962 | 35.85 | 33.62 | 38.19 | 0.04 | 38.15 | - |
| --- | --- | --- | --- | --- | --- | --- | --- | --- | --- | --- | --- | --- | --- | --- |
| EMPALME | Coast | GUAYAS | 124 | 12.73 | 10.59 | 15.17 | 0.01 | 200 | 20.53 | 17.78 | 23.58 | 0.02 | 38.15 | L-L |
| EL TRIUNFO | Coast | GUAYAS | 101 | 17.06 | 13.89 | 20.73 | 0.02 | 151 | 25.5 | 21.6 | 29.91 | 0.03 | 38.15 | - |
| MILAGRO | Coast | GUAYAS | 21 | 1.1 | 0.68 | 1.68 | 0 | 175 | 9.17 | 7.86 | 10.64 | 0.01 | 38.15 | L-L |
| NARANJAL | Coast | GUAYAS | 128 | 13.95 | 11.64 | 16.59 | 0.01 | 339 | 36.95 | 33.12 | 41.1 | 0.04 | 38.15 | L-L |
| NARANJITO | Coast | GUAYAS | 28 | 6.83 | 4.54 | 9.88 | 0.01 | 122 | 29.77 | 24.72 | 35.55 | 0.03 | 38.15 | L-L |
| PALESTINA | Coast | GUAYAS | 2 | 1.02 | 0.12 | 3.7 | 0 | 4 | 2.05 | 0.56 | 5.24 | 0 | 38.15 | - |
| PEDRO CARBO | Coast | GUAYAS | 36 | 6.04 | 4.23 | 8.36 | 0.01 | 214 | 35.88 | 31.23 | 41.02 | 0.04 | 38.15 | - |
| SAMBORONDON | Coast | GUAYAS | 136 | 18.78 | 15.76 | 22.22 | 0.02 | 168 | 23.2 | 19.83 | 26.99 | 0.02 | 38.15 | L-L |
| SANTA LUCIA | Coast | GUAYAS | 23 | 4.94 | 3.13 | 7.42 | 0.01 | 189 | 40.63 | 35.04 | 46.85 | 0.04 | 38.15 | L-L |
| SALITRE | Coast | GUAYAS | 56 | 7.59 | 5.74 | 9.86 | 0.01 | 158 | 21.42 | 18.21 | 25.04 | 0.02 | 38.15 | - |
| SAN JACINTO DE YAGUACHI | Coast | GUAYAS | 10 | 1.23 | 0.59 | 2.25 | 0 | 142 | 17.4 | 14.65 | 20.51 | 0.02 | 38.15 | L-L |
| PLAYAS | Coast | GUAYAS | 187 | 31.76 | 27.38 | 36.66 | 0.03 | 280 | 47.56 | 42.15 | 53.47 | 0.05 | 38.15 | - |
| SIMON BOLIVAR | Coast | GUAYAS | 4 | 1.26 | 0.34 | 3.22 | 0 | 66 | 20.73 | 16.03 | 26.37 | 0.02 | 38.15 | - |
| CRNEL. MARCELINO MARIDUEÑA | Coast | GUAYAS | 0 | 0 | 0 | 2.93 | 0 | 16 | 12.71 | 7.26 | 20.64 | 0.01 | 38.15 | L-L |
| LOMAS DE SARGENTILLO | Coast | GUAYAS | 3 | 1.26 | 0.26 | 3.68 | 0 | 24 | 10.07 | 6.45 | 14.98 | 0.01 | 38.15 | L-L |
| NOBOL | Coast | GUAYAS | 2 | 0.78 | 0.09 | 2.8 | 0 | 29 | 11.24 | 7.53 | 16.14 | 0.01 | 38.15 | L-L |
| GNRAL. ANTONIO ELIZALDE | Coast | GUAYAS | 6 | 4.31 | 1.58 | 9.38 | 0 | 44 | 31.61 | 22.97 | 42.43 | 0.03 | 38.15 | - |
| ISIDRO AYORA | Coast | GUAYAS | 6 | 3.98 | 1.46 | 8.66 | 0 | 29 | 19.23 | 12.88 | 27.62 | 0.02 | 38.15 | L-L |
| LOJA | Highlands | LOJA | 302 | 12.6 | 11.22 | 14.11 | 0.01 | 893 | 37.27 | 34.86 | 39.79 | 0.04 | 44.45 | - |
| CALVAS | Highlands | LOJA | 77 | 23.83 | 18.81 | 29.79 | 0.02 | 70 | 21.67 | 16.89 | 27.37 | 0.02 | 44.45 | - |
| CATAMAYO | Highlands | LOJA | 81 | 24.6 | 19.53 | 30.57 | 0.02 | 86 | 26.12 | 20.89 | 32.25 | 0.03 | 44.45 | - |
| CELICA | Highlands | LOJA | 5 | 2.78 | 0.9 | 6.49 | 0 | 78 | 43.41 | 34.31 | 54.17 | 0.04 | 44.45 | L-L |
| CHAGUARPAMBA | Highlands | LOJA | 4 | 5.35 | 1.46 | 13.71 | 0.01 | 6 | 8.03 | 2.95 | 17.48 | 0.01 | 44.45 | - |
| ESPINDOLA | Highlands | LOJA | 20 | 11.88 | 7.26 | 18.35 | 0.01 | 137 | 81.4 | 68.34 | 96.23 | 0.08 | 44.45 | - |
| GONZANAMA | Highlands | LOJA | 17 | 13.26 | 7.72 | 21.23 | 0.01 | 57 | 44.46 | 33.67 | 57.61 | 0.04 | 44.45 | - |
| MACARA | Highlands | LOJA | 5 | 2.34 | 0.76 | 5.47 | 0 | 42 | 19.68 | 14.18 | 26.6 | 0.02 | 44.45 | - |
| PALTAS | Highlands | LOJA | 79 | 29.36 | 23.24 | 36.59 | 0.03 | 110 | 40.88 | 33.6 | 49.27 | 0.04 | 44.45 | - |
| PUYANGO | Highlands | LOJA | 24 | 14.04 | 9 | 20.9 | 0.01 | 42 | 24.58 | 17.71 | 33.22 | 0.02 | 44.45 | - |
| SARAGURO | Highlands | LOJA | 92 | 23.7 | 19.1 | 29.06 | 0.02 | 131 | 33.75 | 28.21 | 40.04 | 0.03 | 44.45 | - |

| SOZORANGA | Highlands | LOJA | 2 | 2.07 | 0.25 | 7.49 | 0 | 15 | 15.54 | 8.7 | 25.64 | 0.02 | 44.45 | - |
| --- | --- | --- | --- | --- | --- | --- | --- | --- | --- | --- | --- | --- | --- | --- |
| ZAPOTILLO | Highlands | LOJA | 10 | 6.25 | 3 | 11.5 | 0.01 | 26 | 16.26 | 10.62 | 23.82 | 0.02 | 44.45 | - |
| QUILANGA | Highlands | LOJA | 2 | 4.81 | 0.58 | 17.37 | 0.01 | 9 | 21.63 | 9.89 | 41.07 | 0.02 | 44.45 | - |
| OLMEDO | Highlands | LOJA | 8 | 16.1 | 6.95 | 31.72 | 0.02 | 46 | 92.56 | 67.76 | 123.46 | 0.09 | 44.45 | - |
| BABAHOYO | Coast | LOS RIOS | 281 | 15.92 | 14.11 | 17.89 | 0.02 | 1050 | 59.49 | 55.94 | 63.2 | 0.06 | 56.52 | - |
| BABA | Coast | LOS RIOS | 44 | 8.54 | 6.2 | 11.46 | 0.01 | 239 | 46.37 | 40.68 | 52.64 | 0.05 | 56.52 | - |
| MONTALVO | Coast | LOS RIOS | 44 | 16.52 | 12 | 22.17 | 0.02 | 300 | 112.61 | 100.23 | 126.1 | 0.11 | 56.52 | - |
| PUEBLOVIEJO | Coast | LOS RIOS | 71 | 14.4 | 11.25 | 18.17 | 0.01 | 198 | 40.16 | 34.76 | 46.16 | 0.04 | 56.52 | - |
| QUEVEDO | Coast | LOS RIOS | 187 | 8.99 | 7.75 | 10.37 | 0.01 | 783 | 37.63 | 35.04 | 40.36 | 0.04 | 56.52 | - |
| URDANETA | Coast | LOS RIOS | 49 | 13.83 | 10.23 | 18.28 | 0.01 | 261 | 73.67 | 65 | 83.17 | 0.07 | 56.52 | - |
| VENTANAS | Coast | LOS RIOS | 94 | 11.06 | 8.94 | 13.54 | 0.01 | 428 | 50.37 | 45.71 | 55.38 | 0.05 | 56.52 | - |
| VINCES | Coast | LOS RIOS | 74 | 8.41 | 6.61 | 10.56 | 0.01 | 351 | 39.9 | 35.84 | 44.31 | 0.04 | 56.52 | L-L |
| PALENQUE | Coast | LOS RIOS | 39 | 13.08 | 9.3 | 17.88 | 0.01 | 134 | 44.95 | 37.66 | 53.24 | 0.04 | 56.52 | L-L |
| BUENA FE | Coast | LOS RIOS | 46 | 5.39 | 3.95 | 7.2 | 0.01 | 161 | 18.88 | 16.08 | 22.03 | 0.02 | 56.52 | - |
| VALENCIA | Coast | LOS RIOS | 11 | 1.92 | 0.96 | 3.43 | 0 | 115 | 20.04 | 16.54 | 24.05 | 0.02 | 56.52 | - |
| MOCACHE | Coast | LOS RIOS | 16 | 3.24 | 1.85 | 5.26 | 0 | 63 | 12.76 | 9.8 | 16.32 | 0.01 | 56.52 | - |
| QUINSALOMA | Coast | LOS RIOS | 26 | 11.97 | 7.82 | 17.54 | 0.01 | 143 | 65.84 | 55.49 | 77.56 | 0.07 | 56.52 | - |
| PORTOVIEJO | Coast | MANABI | 292 | 9.85 | 8.76 | 11.05 | 0.01 | 1880 | 63.44 | 60.6 | 66.37 | 0.06 | 49.92 | - |
| BOLIVAR | Coast | MANABI | 62 | 13.78 | 10.56 | 17.66 | 0.01 | 208 | 46.22 | 40.15 | 52.95 | 0.05 | 49.92 | - |
| CHONE | Coast | MANABI | 156 | 10.59 | 9 | 12.39 | 0.01 | 522 | 35.45 | 32.48 | 38.63 | 0.04 | 49.92 | - |
| EL CARMEN | Coast | MANABI | 116 | 10.18 | 8.41 | 12.21 | 0.01 | 216 | 18.95 | 16.51 | 21.66 | 0.02 | 49.92 | - |
| FLAVIO ALFARO | Coast | MANABI | 14 | 4.37 | 2.39 | 7.33 | 0 | 34 | 10.62 | 7.35 | 14.83 | 0.01 | 49.92 | - |
| JIPIJAPA | Coast | MANABI | 214 | 27.92 | 24.31 | 31.93 | 0.03 | 450 | 58.72 | 53.42 | 64.4 | 0.06 | 49.92 | - |
| JUNIN | Coast | MANABI | 20 | 9.89 | 6.04 | 15.27 | 0.01 | 164 | 81.07 | 69.14 | 94.47 | 0.08 | 49.92 | - |
| MANTA | Coast | MANABI | 141 | 5.51 | 4.64 | 6.5 | 0.01 | 765 | 29.91 | 27.82 | 32.1 | 0.03 | 49.92 | - |
| MONTECRISTI | Coast | MANABI | 58 | 6.55 | 4.97 | 8.47 | 0.01 | 259 | 29.25 | 25.8 | 33.04 | 0.03 | 49.92 | - |
| PAJAN | Coast | MANABI | 399 | 83.6 | 75.59 | 92.21 | 0.08 | 248 | 51.96 | 45.69 | 58.84 | 0.05 | 49.92 | - |
| PICHINCHA | Coast | MANABI | 77 | 18.08 | 14.27 | 22.6 | 0.02 | 172 | 40.39 | 34.58 | 46.89 | 0.04 | 49.92 | - |
| ROCAFUERTE | Coast | MANABI | 76 | 20.08 | 15.82 | 25.14 | 0.02 | 198 | 52.33 | 45.29 | 60.14 | 0.05 | 49.92 | - |
| SANTA ANA | Coast | MANABI | 25 | 4.49 | 2.9 | 6.62 | 0 | 145 | 26.03 | 21.96 | 30.63 | 0.03 | 49.92 | - |
| SUCRE | Coast | MANABI | 47 | 7.49 | 5.5 | 9.96 | 0.01 | 143 | 22.79 | 19.21 | 26.85 | 0.02 | 49.92 | - |
| TOSAGUA | Coast | MANABI | 77 | 17.99 | 14.2 | 22.49 | 0.02 | 194 | 45.33 | 39.17 | 52.17 | 0.05 | 49.92 | - |
| 24 DE MAYO | Coast | MANABI | 37 | 10.92 | 7.69 | 15.05 | 0.01 | 101 | 29.81 | 24.28 | 36.22 | 0.03 | 49.92 | - |

| PEDERNALES | Coast | MANABI | 79 | 9.4 | 7.44 | 11.72 | 0.01 | 480 | 57.12 | 52.13 | 62.47 | 0.06 | 49.92 | - |
| --- | --- | --- | --- | --- | --- | --- | --- | --- | --- | --- | --- | --- | --- | --- |
| OLMEDO | Coast | MANABI | 8 | 6.66 | 2.88 | 13.13 | 0.01 | 46 | 38.3 | 28.04 | 51.09 | 0.04 | 49.92 | L-L |
| PUERTO LOPEZ | Coast | MANABI | 105 | 36.13 | 29.55 | 43.74 | 0.04 | 219 | 75.36 | 65.71 | 86.03 | 0.07 | 49.92 | - |
| JAMA | Coast | MANABI | 26 | 8.38 | 5.47 | 12.28 | 0.01 | 116 | 37.38 | 30.89 | 44.84 | 0.04 | 49.92 | - |
| JARAMIJO | Coast | MANABI | 50 | 19.21 | 14.26 | 25.32 | 0.02 | 219 | 84.13 | 73.36 | 96.05 | 0.08 | 49.92 | - |
| SAN VICENTE | Coast | MANABI | 36 | 13.59 | 9.52 | 18.81 | 0.01 | 107 | 40.39 | 33.1 | 48.81 | 0.04 | 49.92 | - |
| MORONA | Amazon | MORONA SANTIAGO | 54 | 8.39 | 6.3 | 10.95 | 0.01 | 779 | 121.04 | 112.69 | 129.84 | 0.12 | 82.45 | H-L |
| GUALAQUIZA | Amazon | MORONA SANTIAGO | 75 | 28.88 | 22.72 | 36.2 | 0.03 | 638 | 245.67 | 226.97 | 265.49 | 0.24 | 82.45 | - |
| LIMON INDANZA | Amazon | MORONA SANTIAGO | 16 | 10.4 | 5.94 | 16.88 | 0.01 | 80 | 51.98 | 41.22 | 64.7 | 0.05 | 82.45 | H-L |
| PALORA | Amazon | MORONA SANTIAGO | 26 | 24.79 | 16.19 | 36.32 | 0.02 | 184 | 175.41 | 150.98 | 202.66 | 0.17 | 82.45 | H-H |
| SANTIAGO | Amazon | MORONA SANTIAGO | 19 | 13.46 | 8.1 | 21.01 | 0.01 | 226 | 160.06 | 139.87 | 182.34 | 0.16 | 82.45 | H-L |
| SUCUA | Amazon | MORONA SANTIAGO | 52 | 18.49 | 13.81 | 24.24 | 0.02 | 506 | 179.88 | 164.55 | 196.26 | 0.18 | 82.45 | H-L |
| HUAMBOYA | Amazon | MORONA SANTIAGO | 37 | 20.42 | 14.38 | 28.15 | 0.02 | 414 | 228.48 | 207 | 251.58 | 0.22 | 82.45 | H-H |
| SAN JUAN BOSCO | Amazon | MORONA SANTIAGO | 35 | 57.1 | 39.77 | 79.41 | 0.05 | 137 | 223.49 | 187.64 | 264.2 | 0.21 | 82.45 | H-H |
| TAISHA | Amazon | MORONA SANTIAGO | 554 | 128.3 | 117.84 | 139.44 | 0.13 | 945 | 218.85 | 205.12 | 233.26 | 0.22 | 82.45 | H-H |
| LOGROÑO | Amazon | MORONA SANTIAGO | 45 | 42.53 | 31.02 | 56.91 | 0.04 | 337 | 318.53 | 285.42 | 354.42 | 0.31 | 82.45 | H-H |
| PABLO SEXTO | Amazon | MORONA SANTIAGO | 34 | 97.98 | 67.86 | 136.92 | 0.08 | 105 | 302.59 | 247.49 | 366.31 | 0.27 | 82.45 | H-H |
| TIWINTZA | Amazon | MORONA SANTIAGO | 4 | 2.9 | 0.79 | 7.44 | 0 | 161 | 116.92 | 99.56 | 136.44 | 0.11 | 82.45 | H-L |
| ZAMORA | Amazon | ZAMORA CHINCHIPE | 38 | 12.63 | 8.94 | 17.34 | 0.01 | 89 | 29.59 | 23.76 | 36.41 | 0.03 | 49.62 | - |
| CHINCHIPE | Amazon | ZAMORA CHINCHIPE | 18 | 15.06 | 8.93 | 23.81 | 0.02 | 73 | 61.09 | 47.88 | 76.81 | 0.06 | 49.62 | - |
| NANGARITZA | Amazon | ZAMORA CHINCHIPE | 8 | 9.08 | 3.92 | 17.89 | 0.01 | 27 | 30.65 | 20.2 | 44.59 | 0.03 | 49.62 | - |
| YACUAMBI | Amazon | ZAMORA CHINCHIPE | 13 | 13.39 | 7.13 | 22.89 | 0.01 | 37 | 38.11 | 26.83 | 52.52 | 0.04 | 49.62 | - |
| YANTZAZA | Amazon | ZAMORA CHINCHIPE | 67 | 24.68 | 19.12 | 31.34 | 0.02 | 153 | 56.35 | 47.78 | 66.02 | 0.06 | 49.62 | H-H |
| EL PANGUI | Amazon | ZAMORA CHINCHIPE | 36 | 26.59 | 18.62 | 36.81 | 0.03 | 77 | 56.87 | 44.88 | 71.08 | 0.06 | 49.62 | H-H |
| CENTINELA DEL CONDOR | Amazon | ZAMORA CHINCHIPE | 6 | 6.47 | 2.37 | 14.07 | 0.01 | 42 | 45.26 | 32.62 | 61.18 | 0.04 | 49.62 | - |
| PALANDA | Amazon | ZAMORA CHINCHIPE | 7 | 5.94 | 2.39 | 12.24 | 0.01 | 55 | 46.69 | 35.17 | 60.77 | 0.05 | 49.62 | - |
| PAQUISHA | Amazon | ZAMORA CHINCHIPE | 43 | 71.91 | 52.04 | 96.86 | 0.07 | 82 | 137.12 | 109.06 | 170.21 | 0.13 | 49.62 | - |
| SANTA ELENA | Coast | SANTA ELENA | 884 | 43.85 | 41.01 | 46.84 | 0.04 | 923 | 45.79 | 42.88 | 48.84 | 0.05 | 41.96 | - |
| SALINAS | Coast | SANTA ELENA | 265 | 28.58 | 25.24 | 32.24 | 0.03 | 297 | 32.03 | 28.49 | 35.89 | 0.03 | 41.96 | - |
| NABON | Highlands | AZUAY | 71 | 38.5 | 30.07 | 48.57 | 0.04 | 63 | 34.16 | 26.25 | 43.71 | 0.03 | 28.1 | - |
| LA LIBERTAD | Coast | SANTA ELENA | 152 | 11.74 | 9.95 | 13.77 | 0.01 | 154 | 11.9 | 10.09 | 13.93 | 0.01 | 41.96 | - |
| PINDAL | Highlands | LOJA | 6 | 5.64 | 2.07 | 12.27 | 0.01 | 31 | 29.14 | 19.8 | 41.36 | 0.03 | 44.45 | - |
| ARENILLAS | Coast | EL ORO | 20 | 6.72 | 4.1 | 10.38 | 0.01 | 59 | 19.82 | 15.09 | 25.56 | 0.02 | 28.8 | - |

| HUAQUILLAS | Coast | EL ORO | 23 | 4.01 | 2.54 | 6.02 | 0 | 43 | 7.5 | 5.43 | 10.11 | 0.01 | 28.8 | - |
| --- | --- | --- | --- | --- | --- | --- | --- | --- | --- | --- | --- | --- | --- | --- |
